# Synovial Sarcoma of the Hand

**DOI:** 10.1155/2020/8491864

**Published:** 2020-06-05

**Authors:** Hovsep Ohan, Greg Minassian, Asaad H. Samra, Matthew J. Zdilla

**Affiliations:** ^1^Department of Pathology, Anatomy and Laboratory Medicine (PALM), Robert C. Byrd Health Sciences Center, West Virginia University School of Medicine, Morgantown, West Virginia, USA; ^2^Rutgers New Jersey Medical School, Newark, New Jersey, USA; ^3^Hackensack Meridian Health, Holmdel, New Jersey, USA; ^4^Department of Natural Sciences and Mathematics, West Liberty University, West Liberty, West Virginia, USA; ^5^Department of Graduate Health Sciences, West Liberty University, West Liberty, West Virginia, USA

## Abstract

The incidence of synovial sarcoma is 1.548 per 1,000,000. Synovial sarcoma localized to the palmar surface should, therefore, be considered extremely rare. This report documents a 34-year-old male with a right hand mass that had been present for a few years, continuing to grow in size. The mass was located at the palm and extended from the mid-third metacarpal to involve all digits except the thumb. The mass was determined to be monophasic synovial sarcoma on histopathologic exam. Fluorescence in situ hybridization for SYT gene rearrangement was positive in 72% of cells. Resection of the mass was followed by radiation and chemotherapy. The patient had a long-term follow-up of 3.5 years with no evidence of any local recurrence of the tumor. This report increases awareness of this extremely rare malignancy—an awareness that is crucial for early diagnosis and improved survival rates. It is more common at younger ages but it can occur at any age, so it should be suspected and included in the differential diagnosis, especially when evaluating slow growing, nonresolving hand lesions.

## 1. Introduction

The incidence of synovial sarcoma, in general, is 1.548 per 1,000,000 [1]. Synovial sarcoma localized to the palmar surface should, therefore, be considered extremely rare [2]. In the unusual event in which sarcoma has been identified in the palmar aspect of the hand, the incidence of digit involvement is less common than that of the carpus [2].

Because of the rarity of synovial sarcoma of the hand, as well as a paucity of reports on such pathology, its identification warrants a detailed account. Therefore, this report documents a rare case of synovial sarcoma existing in the digits of a 34-year-old man.

## 2. Case Presentation

The written consent of the individual described in this study was obtained.

A 34-year-old male with an unremarkable medical history presented with a chief compliant of what he believed to be a cyst on the palmar side of his right hand. The patient stated that the mass had been there for a few years. The area had grown in size and had been causing griping issues. He also complained of numbness at the finger tips which he attributed to the mass.

**Figure 1:**
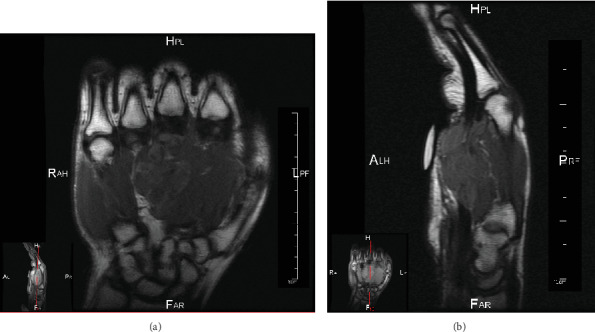
T1-weighted coronal and sagittal MRI revealing a large, lobulated, soft tissue lesion with aggressive features located on the ventral surface of the hand and extending from the mid-third digit to the fifth digit. (a) Coronal view demonstrating a hypointense enhancing lobular lesion at the third ray. (b) Sagittal view showing a soft tissue lesion surrounding the flexor tendons and extending into the interspace between the head of the third and fourth metacarpals.

Physical examination revealed a soft, nontender, nonpulsatile mass, and normal blood flow to the finger tips. Magnetic resonance imaging, performed with contrast, demonstrated a large lobulated enhancing lesion measuring 4.7 cm in its greatest dimension (Figure 1). The lesion extended from the mid-third metacarpal ventrally, surrounding the dorsal aspect of the flexor tendons and involved the second, third, fourth, and partially, the fifth digits. The bone marrow signal was unremarkable, thereby indicating a lack of bone involvement by the tumor.

**Figure 2:**
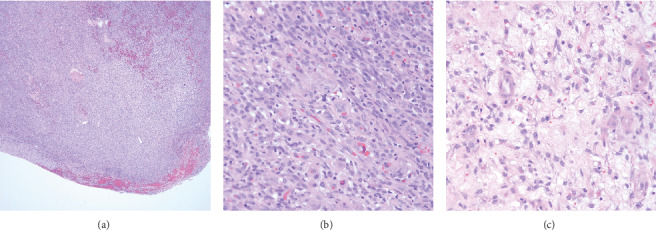
Histologic evaluation of a hand tumor indicative of monophasic synovial sarcoma. (a) At low power magnification, a well-circumscribed hypercellular mass with areas of fibrosis and high vascularity can be seen. **(b)** The monophasic synovial sarcoma shows a densely packed, interlacing spindled cell proliferation with monomorphic cells arranged in a myxoid stroma. The nuclei are ovoid and pale-staining with small nucleoli. Rich vascularity is present. (c) High-magnification revealing mitotic figures.

Excisional biopsy of the mass revealed a soft-tissue tumor with ovoid spindle cells surrounding hyalinized material without gross or histologic evidence of hemorrhage or necrosis. Occasional mitotic figures were identified (Figure 2). The lesion appeared to have both hyper- and hypocellular areas. Immunostaining was positive for beta-catenin and vimentin. Immunostaining was negative for smooth muscle actin, desmin, CD31, CD34, CD68, and S100.

Complete resection of the mass was done with R0 margins. The resected tissue was characterized by a well-circumscribed mass that involved deep soft tissue and skeletal muscle without involvement of underlying bone. The tumor showed hyalinization, fibrosis, and focal calcification. The specimen consisted of one pale pink unoriented ellipse of skin measuring 2 × 1.8 × .5 cm. The epidermal surface showed one brown nodular lesion measuring 1.8 × 1.5 × cm. Histopathologic exam showed skin with focal ulceration and associated granulation tissue overlying a cellular mesenchymal neoplasm composed of monomorphic spindle cells arranged in compact fascicles admixed with coarse collagen bundles, myxoid stroma, and scattered mast cells. Accordingly, the tumor was classified as a monophasic synovial sarcoma (Figure 2). Because of the histologic appearance, a request was made for fluorescence in situ hybridization for SYT gene rearrangement, which was positive in 72% of cells indicating the presence of chromosome t (X; 18) or its variant involving the SYT gene commonly associated with synovial sarcoma. In addition to FISH testing, a request was made for Bcl-2 and CD99 immunostaining; however, because the diagnosis was confirmed with the SYT genetic rearrangement with FISH, further immunostaining was deemed unnecessary.

Surgery was followed by adjuvant radiation treatment and three cycles of chemotherapy with Adriamycin and Ifosfamide. Reconstructive surgery was deemed unnecessary. The patient had regular follow-ups for three and a half years. At the last follow-up, the patient noted pain when trying to write or type for any extended period of time. Also, there were deficiencies in range of motion related to scar tissue from radiation. There was no evidence of any local recurrence of the tumor.

## 3. Discussion

Soft tissue sarcomas of the upper extremities are rare with perhaps only one or two undiagnosed soft tissue sarcomas encountered by hand surgeons throughout their entire career [3]. The most common site of soft tissue sarcoma is at the lower extremity (60%) (with tendency for the knee, ankle, and hip), followed by the upper extremity (23%) (with tendency for the shoulder), and the head and neck (9%) [1, 2, 4]. Further, it tends to be located close to the large joints of the extremities, especially the knee and ankle. A synovial sarcoma in the palmar aspect of the hand which involves the digits is extremely rare and, therefore, particularly noteworthy.

There may be a long delay in diagnosis, or misdiagnosis, altogether, due to slow growth pattern, varied radiological features, change in size of the tumor, and joint pain which can mimic traumatic pain [5]. Therefore, synovial sarcoma cases may be initially suspected to be myositis, hematoma, synovitis, tendonitis, bursitis, abscess, and hematoma; therefore, delay in diagnosis is common [6].

A unique chromosomal translocation t (X; 18), (p11; q11), as in our case, involving genes SS18 and either SSX1, SSX2, or SSX4, is involved in the oncogenesis of synovial sarcoma [7]. Synovial sarcoma is a mesenchymal spindle cell tumor which displays variable epithelial differentiation, is of unknown histogenesis, and is unrelated to synovium. Four morphologic variants have been described: classic biphasic type, monophasic fibrous type (the most common variant, as in our case), monophasic epithelial type, and poorly differentiated type [8]. Immunohistochemically, Bcl-2 protein expression has been described as a characteristic marker and is useful for its differentiation from other sarcomas. Cytokeratin and CD99 are also used to detect it [2, 8].

Although radiologic features of these tumors are not pathognomonic, cross-sectional imaging features are vital for staging tumor extent and planning surgical resection [9]. For localized non-high-risk disease, treatment consists of surgical resection with wide margin combined with adjuvant radiotherapy [1, 10]. With regard to high-risk disease of extremity and chest wall, adjuvant combination chemotherapy might be considered [5, 10]. In about 50% of cases, metastasis occurs mostly in the lungs; combination treatment with doxorubicin and ifosfamide is a preferred option [10].

The prognosis of primary, nonmetastasized disease is related to the age of the patient, with much better relative survival in children compared to older patients, and more genomic instability with increasing age [11]; whereas, histologic grading is the best indicator of metastasis outcome in adult soft tissue sarcoma, which consists of 3-grade systems based on histologic type, tumor necrosis, and mitotic activity [12]. Significant factors affecting overall survival are age at diagnosis, sex, tumor localization, and tumor size with a five-year overall survival for all synovial sarcomas at 60.5% [1, 13]. The survival rates were unimproved across three decades in a large sample studied [1].

A reported case of a 63-year-old woman with a hand resected synovial sarcoma with no metastases followed by radiotherapy and chemotherapy with 12-year disease-free period, came back with local recurrence and died within one year of recurrence, whereas another reported case of a 22-year-old female with local recurrences occurring twice after resection within 10 years and without any metastases, indicating a potential influence of age at presentation upon survival [2, 6]. Our case shows a young adult with wide resection of well-circumscribed mass without any metastasis with long-term follow-up showing no evidence of local recurrence.

In conclusion, synovial sarcoma of the hand is a rare, yet highly malignant type of soft tissue sarcoma, for which survival has not improved significantly during the past three decades [1]. This report increases health-care providers' awareness of this extremely rare malignancy—an awareness that is crucial for early diagnosis and improved survival rates. It is more common at younger ages but it can occur at any age, so it should be suspected and included in the differential diagnosis, especially when evaluating slow growing, nonresolving hand lesions.
